# Screening for type 2 diabetes is feasible, acceptable, but associated with increased short-term anxiety: A randomised controlled trial in British general practice

**DOI:** 10.1186/1471-2458-8-350

**Published:** 2008-10-07

**Authors:** Paul Park, Rebecca K Simmons, A Toby Prevost, Simon J Griffin

**Affiliations:** 1 General Practice & Primary Care Research Unit, Department of Public Health and Primary Care, University of Cambridge, Cambridge, UK; 2 MRC Epidemiology Unit, Institute of Metabolic Science, Cambridge, UK

## Abstract

**Background:**

To assess the feasibility and uptake of a diabetes screening programme; to examine the effects of invitation to diabetes screening on anxiety, self-rated health and illness perceptions.

**Methods:**

Randomised controlled trial in two general practices in Cambridgeshire. Individuals aged 40–69 without known diabetes were identified as being at high risk of having undiagnosed type 2 diabetes using patient records and a validated risk score (n = 1,280). 355 individuals were randomised in a 2 to 1 ratio into non-invited (n = 238) and invited (n = 116) groups. A stepwise screening programme confirmed the presence or absence of diabetes. Six weeks after the last contact (either test or invitation), a questionnaire was sent to all participants, including non-attenders and those who were not originally invited. Outcome measures included attendance, anxiety (short-form Spielberger State Anxiety Inventory-STAI), self-rated health and diabetes illness perceptions.

**Results:**

95 people (82% of those invited) attended for the initial capillary blood test. Six individuals were diagnosed with diabetes. Invited participants were more anxious than those not invited (37.6 vs. 34.1 STAI, p-value = 0.015), and those diagnosed with diabetes were considerably more anxious than those classified free of diabetes (46.7 vs. 37.0 STAI, p-value = 0.031). Non-attenders had a higher mean treatment control sub-scale (3.87 vs. 3.56, p-value = 0.016) and a lower mean emotional representation sub-scale (1.81 vs. 2.68, p-value = 0.001) than attenders. No differences in the other five illness perception sub-scales or self-rated health were found.

**Conclusion:**

Screening for type 2 diabetes in primary care is feasible but may be associated with higher levels of short-term anxiety among invited compared with non-invited participants.

**Trial registration:**

ISRCTN99175498

## Background

Type 2 diabetes meets many of the criteria for suitability for screening. It is increasingly common and creates a substantial burden of suffering and health service use [1]. (However, there is continuing uncertainty concerning feasibility, uptake and the overall benefits and costs, both economic and psychological [2,3]. Even a relatively small disbenefit to the majority of people who screen negative, either directly or through false reassurance, may outweigh significant benefits to the minority whose condition is detected and treated earlier. Consequently it is important to quantify adverse effects of screening at the population level prior to initiation of a programme [3]. However, such adverse effects are rarely assessed using randomised trial designs.

Descriptions of screening programmes for diabetes have highlighted possible harmful effects of misdiagnosis among screened individuals [4], negative attitudes among patients diagnosed through screening [5] and a small reduction in perceived health after a false positive result in a screening test for gestational diabetes [6]. However, data from observational studies among screened patients suggest that psychological harms associated with screening are small and short-lived [7,8]. Furthermore, in a recent large controlled trial which compared screened and unscreened individuals, the few significant differences were not considered clinically important [9].

This study reports results from an *individually randomised *controlled trial undertaken during the pilot phase of the ADDITION trial of a stepwise screening programme for diabetes [10] (NCT 00237549). We aimed to examine (i) the feasibility of a stepwise screening programme in general practice; (ii) the uptake of the screening programme; (iii) the effects of the programme on participant's anxiety, self-rated health and illness perceptions of diabetes.

## Methods

**Figure 1:**
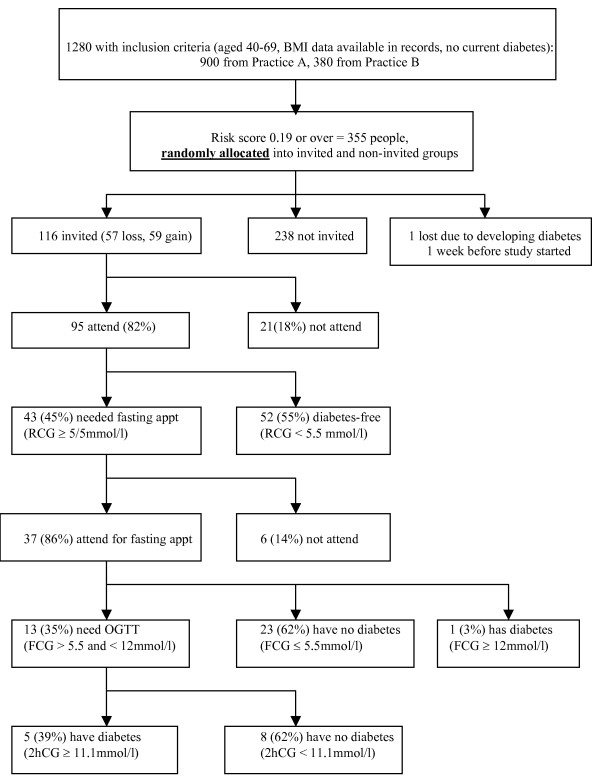
Flow chart of participant attendance and outcome of screening.

This RCT was undertaken as part of the pilot phase of the ADDITION trial [10]. However, none of the practices or participants in the randomised trial reported in this paper were included in the main ADDITION study [9,10]. Two general practices in Cambridgeshire, East of England, participated. Practice A is a rural practice in a market town near Huntingdon with a list of 7,800 patients. Practice B has 9,537 patients in central Cambridge. Both practices serve relatively affluent populations and receive few deprivation payments. Eligible participants were aged 40–69 without known diabetes, identified as being at high risk of having undiagnosed type 2 diabetes using a score based on information routinely recorded in electronic patient records (age, sex, BMI and prescribed medication) [11]. The complete data required to calculate the diabetes risk score were recorded in the practice computer files for 3,792 of the 5,844 people (65%) who met the inclusion criteria. We randomly selected 1,280 participants from this group using SPSS (v.9.0.1) and those falling above a diabetes risk score threshold of 0.19 comprised the study sample (n = 355). Using SPSS, the study sample was then individually randomised in a 2 to 1 ratio into non-invited (238) and invited (116) groups, to optimise study power and feasibility. The invited group was further randomised to receive loss (57) or gain (59) framed invitations by personal letter [12]. A flow chart of attendance is shown in Figure 1.

### Diabetes screening

Those who attended their screening appointment underwent a step-wise procedure to confirm the presence or absence of diabetes. In the first stage, a random capillary blood sample was taken by the practice nurse and tested using HemoCue B-Glucose glucometers (HemoCue Ltd., Angelholm, Sweden). Participants with a random capillary glucose result ≥ 5.5 mmol/l were classified as screening-test-positive and were asked to re-attend for a fasting capillary blood glucose test at a later date. Individuals whose random capillary glucose result was < 5.5 mmol/l were classified as not having type 2 diabetes and were informed of their result. In the second stage, participants whose fasting capillary glucose result was ≥ 12 mmol/l were informed that they had type 2 diabetes. Participants with a fasting result ≤ 5.5 mmol/l were informed that they did not have diabetes. Those participants with a fasting glucose of 5.5 – 12 mmol/l were classified as requiring a further confirmatory 75 g oral glucose tolerance test, which was undertaken at the same visit. Those whose 2-hour capillary glucose was ≥ 11.1 mmol/l were informed that they had type 2 diabetes. Those who tested positive were informed of the consequences of the diagnosis of diabetes in a subsequent consultation with their general practitioner. The thresholds for fasting and 2-hour glucose in this study were those recommended by the World Health Organisation in 1999 for diagnosis using capillary glucose testing [13]. A flow chart of the step-wise screening procedure is shown in Figure 1.

Six weeks after the last contact (either test or invitation), a questionnaire assessing anxiety, self-rated health and illness perceptions was sent to all participants, including non-attenders and those who were not originally invited. A freepost return envelope was included, and repeat duplicate questionnaires were sent to non-responders at 4 and 8 weeks following the initial mailing.

### Questionnaire measurements

Anxiety was measured using the short form of the Spielberger State Anxiety Inventory (STAI). This consists of six, 4-point Likert scales, and has been shown to correlate well (r > 0.9) with the results of the longer questionnaire from which it was derived [14], which is in turn associated with indicators of anxiety [15]. The STAI used in this study measures state (current state of anxiety or mood) rather than trait (recurring or individual characteristics of anxiety or mood) anxiety [14]. Participants were also asked about their self-perceived health with a single, 5-point Likert scale (excellent, very good, good, fair, poor), previously used in a study of screening for gestational diabetes [6]. Illness perceptions were assessed with the 50-item diabetes Illness Perception Questionnaire (IPQ). It was derived from the most accurate measure of illness perceptions available at the time [16,17] and has shown good internal consistency and test-retest reliability in its various components [18]. The questionnaire was based on Leventhal's self-regulatory model of cognitive psychology, which predicts how patients form their own personal models of their disease (illness perceptions) to make sense of their illness experience and help them form coping responses [19,20]. The IPQ expands the five basic components of illness representations [20] into the following sub-components:

1. Identity (whether certain symptoms are associated with diabetes; e.g. thirst, skin changes)

2. Timeline acute/chronic (e.g. a statement that agrees that diabetes is chronic would be: "diabetes lasts a long time")

3. Timeline cyclical (e.g. "the symptoms of diabetes come and go in cycles")

4. Consequences of diabetes (e.g. "Diabetes has serious financial consequences")

5. Personal control of diabetes (how one's attitude and behaviour affects diabetes, e.g. "The course of diabetes depends on the patient")

6. Treatment control (how medical treatment affects diabetes, e.g. "Treatment can control diabetes")

7. Emotional representation (what emotions are associated with diabetes, e.g. "Diabetes makes me feel afraid.")

8. Causes of diabetes (e.g. being overweight, patient's personality)

### Statistical analysis

The primary outcome, STAI, was scaled to have the same range (20 to 80) as the original longer form. STAI and IPQ scores were assessed for internal consistency using Cronbach's alpha. Unpaired t-tests were used to compare mean scores between groups for state anxiety, illness representations and self-reported health. Attendance rates were summarised with exact 95% confidence intervals. Pearson's chi-squared test was used to compare response rates between groups. Linear regression was used to assess whether STAI was associated with baseline characteristics or other psychological measures. Analyses were completed using SPSS (version 12.0) and StatXact (version 4.0). All tests were two-sided and assessed at the 5% level of statistical significance. 140 non-invited and 70 invited participants responding to the questionnaire (60% response rate), would have provided 80% power to detect a five-point difference in mean STAI (SD = 12) between non-invited and invited arms using a two-sided unpaired t-test at the 5% level of significance (nQuery version 4.0 software).

This trial was approved by Cambridge LREC (00/071). Participants provided written consent.

## Results

Baseline characteristics for invited and non-invited participants are shown in Table 1. Two-thirds were men and one-third women, reflecting the fact that male sex confers an increased risk for having undiagnosed type 2 diabetes. There was no difference in mean age, mean BMI, or the percentage prescribed anti-hypertensive/steroid medication between randomised groups.

**Table 1:** Baseline characteristics by trial group (invited and non-invited participants). All values are means (SD) unless stated otherwise.

**Baseline characteristics**	**Invited (n = 116)**	**Non-invited (n = 238)**
Age (years)	58.3 (7.3)	58.9 (7.2)
Sex (number (%) male)	76 (65.5%)	149 (62.6%)
BMI (kg/m^2^)	31.8 (4.5)	31.3 (4.1)
Number (%) prescribed anti-hypertensive medication	42 (36.2%)	91 (38.2%)
Number (%) prescribed steroid medication	10 (8.6%)	6 (2.5%)

### Screening uptake

95 people attended the initial screening test appointment (82%, 95% CI: 75% to 89%) out of the 116 patients invited. Those who attended did not significantly differ from non-attenders in terms of age, sex, and BMI. However, attenders were more likely to have been prescribed antihypertensive or steroid medication (data not shown). The rate of attendance through the complete step-wise screening process was 77% (95% CI: 68% to 84%).

### Glucose testing (Figure 1)

Of the 95 people who attended for the random capillary glucose test, 43 (45%) had a glucose ≥ 5.5 mmol/l and were asked to re-attend for a fasting appointment. 37 (86%) of these people returned, of which 23 (62%) were declared free of diabetes, 13 (35%) required a 2-hour oral glucose tolerance test, and one person had a fasting glucose > 12 mmol/l and was labelled as having type 2 diabetes. 5 (39%) of those undergoing a 2h-OGTT test had a 2 h glucose of ≥ 11.1 mmol/l and were labelled as having diabetes. In total, 6 individuals (5% (95%CI: 2% to 11%) were given the diagnosis of diabetes as a result of the screening programme.

### Psychological outcomes

The overall response rate to the questionnaire was 68%. There was no difference in baseline characteristics between responders and non-responders, and no difference in the response rate between those invited and not invited to screening. The STAI six-item anxiety scale showed a Cronbach's alpha of 0.84, while the IPQ sub-scales showed a range of internal consistency (0.38 to 0.90). The alpha values were lowest for the timeline cyclical and treatment control sub-scales, with alphas of 0.40 and 0.38 respectively. There was no significant difference in attendance or anxiety between those invited with loss and gain frame invitations (Park, P., Simmons, R.K., Prevost, A.T., Griffin, S.J., *A randomised evaluation of loss and gain frames in an invitation to screening for type 2 diabetes – effects on attendance, anxiety and self-rated health*. In submission, Eval Health Prof, 2008), so they were treated as a single group.

**Figure 2:**
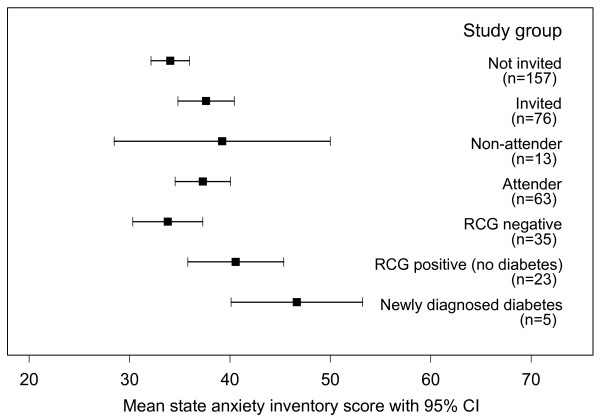
Mean STAI anxiety scores by study group; RCG = random capillary glucose.

Invited participants were significantly more anxious than those not invited (Table 2). No statistical difference was found between attenders and non-attenders (Table 3). Anxiety was higher among those participants who progressed further through the screening programme (Figure 2). Those who were eventually diagnosed with diabetes had the highest STAI values. Individuals with newly diagnosed diabetes (positive test result) had a significantly higher STAI than those who tested negative on the first RCG.

**Table 2:** Comparison of psychological outcomes between invited and non-invited participants. All values are means (SD).

	Invited (n = 77)*	Non-invited (n = 168)*	**p-value from t-test**
STAI anxiety (range 20–80)	37.6 (12.2)	34.1 (12.1)	0.015
			
Self-perceived health; range (1 to 5)**	2.97 (0.86)	2.95 (0.87)	0.82
			
Illness representation subscales; (range 1 to 5)			
- Acute/chronic illness	3.95 (0.48)	4.07 (0.46)	0.07
- Cyclic illness	2.99 (0.42)	2.93 (0.46)	0.64
- Consequences	3.25 (0.47)	3.40 (0.50)	0.08
- Personal consequences	3.67 (0.44)	3.70 (0.45)	0.60
- Treatment control	3.60 (0.41)	3.60 (0.43)	0.87
- Emotional representations	2.58 (0.66)	2.63 (0.75)	0.74
- Illness coherence	3.10 (0.83)	3.05 (0.86)	0.98

* Numbers may vary from those in Figure 1 due to non-response of questionnaires

* *scored 1 = poor 2 = fair 3 = good 4 = very good 5 = excellent

**Table 3:** Comparison of psychological outcomes between attenders and non-attenders for initial glucose screening. All values are means (SD).

	Attenders (n = 64)*	Non-attenders (n = 13)*	**p-value from t-test**
STAI anxiety (range 20–80)	37.3 (10.9)	39.2 (17.8)	0.77
			
Self-perceived health; range (1 to 5)**	2.92 (0.86)	3.23 (0.83)	0.24
			
Illness representation subscales; (range 1 to 5)			
- Acute/chronic illness	3.95 (0.48)	3.94 (0.47)	0.90
- Cyclic illness	2.98 (0.41)	3.07 (0.49)	0.90
- Consequences	3.28 (0.47)	3.09 (0.43)	0.22
- Personal consequences	3.66 (0.43)	3.74 (0.55)	0.74
- Treatment control	3.56 (0.41)	3.87 (0.23)	0.016
- Emotional representations	2.68 (0.61)	1.81 (0.45)	0.001
- Illness coherence	3.07 (0.84)	3.29 (0.81)	0.35

* Numbers may vary from those in Figure 1 due to non-response of questionnaires

**Scored 1 = poor 2 = fair 3 = good 4 = very good 5 = excellent

There were no significant differences between the invited and non-invited groups for self-rated health and IPQ sub-scales. Participants who did not attend the first random glucose test (and therefore did not attend any subsequent testing) differed significantly from those who did attend in two sub-scales of their illness perceptions of diabetes (Table 3). Non-attenders had a higher mean treatment control sub-scale and a lower mean emotional representation sub-scale. There were no other differences in psychological outcomes between attenders and non-attenders, although the number of non-attenders was small.

### Predictors of anxiety

None of the baseline characteristics (age, sex, BMI, prescribed anti-hypertensive/steroid medication) were associated with anxiety (data not shown). Of the psychological outcomes, consequences, emotional representations and illness coherence sub-scales of the IPQ were correlated with raised anxiety. Overall, mean anxiety level was higher among patients in Practice A than Practice B, but this was largely due to the fact that all those labelled with type 2 diabetes were from Practice A (no difference remained when those with diabetes were removed from the analysis).

## Discussion

This study has shown that it is feasible and acceptable to screen for type 2 diabetes in two general practices in Cambridgeshire using a risk score to select a high-risk population and capillary glucose measurement to test for diabetes. This finding was replicated in the ADDITION (Cambridge) controlled trial [9]. There was high attendance for both stages of testing and the methods used to select participants, invite people for screening and test for diabetes all proved acceptable and practical. However, relatively few participants were diagnosed, even in this high risk group. Furthermore, after six weeks, participants who had been invited to screening exhibited modest differences in anxiety compared with those who were not invited, and those few who were diagnosed with diabetes were significantly more anxious than those who attended for the first stage of screening but were classified free of diabetes.

### Attendance

Attenders did not significantly differ from non-attenders, except that they were more likely to have been prescribed either antihypertensive or steroid medication. It is possible that attenders were more likely to have already been labelled with a chronic disease (such as hypertension) and had become used to returning regularly to the practice for monitoring, testing and treatment, and this in turn made them more motivated or less anxious about attending for screening for diabetes. Our results for age and sex contrast with findings from other screening studies where those who attend for health screening are more likely to be female [21] and older [22].

### Anxiety

Being invited to screening was associated with raised anxiety Evidence of causality is strengthened by the apparent dose-response relationship as participants who progressed further through the screening process exhibited higher levels of anxiety compared to those who did not progress so far or were not invited. There was also a significant difference in anxiety between test-positives and those who tested negative at the first stage of screening, suggesting an adverse effect of the label of diabetes. These results were gathered by questionnaire at least 6 weeks after the last appointment, implying that these are medium-term changes in anxiety rather than an immediate short-term effect of the invitation or test.

A systematic review of the psychological impact of being presented with risk information [23] found that anxiety was not significantly raised in the long term (defined by the review as more than one month after the information was presented). Observational studies suggest that there is little psychological harm associated with a diagnosis of diabetes by screening [8]. These findings are supported by data from the ADDITION (Cambridge) controlled trial [9]. The elevated anxiety found in the present study after at least 6 weeks for both test-negatives and test-positives is therefore unexpected. This may be due to the small numbers in the present study, the fact that this study was individually randomised, as opposed to a cohort study or controlled trial with participants allocated by cluster, or the timing of measurement. It is possible that the time-scale in this study is not sufficiently long-term, and that people more used to living with diabetes would become less anxious (response-shift). Qualitative work suggests that this asymptomatic condition is not considered to be serious [24]. However, studies of mood and affect in those with diabetes suggest that the label of diabetes is associated with depression and anxiety in the long term [25,26], as the burden of disease and treatment becomes more salient. The mean anxiety level (STAI score 46.7) in those with newly diagnosed diabetes is equivalent to a clinical diagnosis of anxiety according to ICD-10 (STAI score 42 or more) and similar to that found in pregnant women who have just received an abnormal test result for maternal serum alpha-fetoprotein screening indicating increased foetal risk of spina bifida and Down's syndrome [14]. Some degree of anxiety may be unavoidable and may even have some positive attributes, for example, increasing motivation for behaviour change. However, the level of anxiety associated with screening in this study merits consideration prior to initiation of a screening programme. No statistical difference was found between invited groups after excluding those with diabetes, though increased anxiety was seen in invitees. As with all screening programmes, a key challenge is to ensure that any benefit of early diagnosis and treatment for the few outweighs the population sum of small adverse effects among participating individuals.

Since the results in this study would seem to indicate that being invited for screening is associated with anxiety, it was important to examine which variables were associated with greater anxiety. Only the consequences, emotional representations and illness coherence sub-scales were positively correlated with raised anxiety, which implies that those with raised anxiety had more negative feelings about diabetes and its consequences. The provision of information at the invitation and screening stages about the potential for controlling diabetes and its complications may therefore be useful in reducing anxiety.

### Illness representations

Non-attenders had significantly higher treatment control and lower negative emotional perceptions than attenders. This suggests that non-attenders are not sufficiently concerned about the significance of diabetes risk to attend, for example, they believe that diabetes can be controlled effectively by treatment and/or do not associate diabetes with negative emotions. There were no differences in the results of the IPQ sub-scales between those who were invited and those not invited to screening, implying that invitation to screening and its sequelae did not alter the participants' illness perceptions of diabetes. It is therefore likely that the observed differences between attenders and non-attenders were present at baseline and were not caused by screening, that is to say, the differences in the IPQ sub-scales were predictors rather than consequences of attendance. These findings have implications for the design of future diabetes prevention and invitation materials concerning screening for type 2 diabetes, as previously discussed.

### Strengths and limitations

This was a fairly small trial with a limited set of measures yet had the advantage of being population-based and unlike all previous studies addressing this topic, included individual randomisation to screening or control group. Although we included only two GP practices, the study population was similar to the general population of England and Wales for age, sex and BMI as assessed by the Health Survey for England in 1994 [27], though the participants were largely Caucasian. The study population was not completely representative of the background population of the general practices since participants were selected for having complete data for calculating the risk score, and constituted 65% of the possible eligible population.

We used capillary glucose measurements for diabetes screening and diagnosis which are approved by the WHO [13], but used less frequently in clinical practice than venous plasma measurements. Further, we did not establish the diagnosis of type 2 diabetes with two positive results on separate days, as recommended by the WHO for participants without known symptoms of diabetes [13]. Given the variability of glucose measures [28], this may have had a small effect on the accuracy of diagnostic labels but not the study hypothesis concerning the effects of the invitation or the disease label.

The questionnaire in this study was administered at only one time point and the study design relied on randomisation into invited and non-invited groups to quantify the effects of invitation to screening on the variables measured. This approach has the advantage of avoiding the possible accommodation effect of repeat administration of questionnaires, an issue that may have limited interpretation of an earlier diabetes screening study [29]. Single questionnaire testing is also more convenient for participants, less costly, and associated with higher response than repeat questionnaires. However, the disadvantage of single testing is the inability to assess change within individuals over time. Finally, it is worth considering the questionnaire measurements themselves. For the illness perception questionnaire, it is uncertain how applicable a questionnaire designed for use in a population with a chronic disease is to a mostly disease-free group. Yet we demonstrated that beliefs about a condition may be an important determinant of screening uptake. An early version of the IPQ was used and the sub-scales had mixed internal consistency with Cronbach's alpha values ranging from 0.38 to 0.90. However, it did not differ substantially from the current more widely used version [16]. The low Cronbach's alpha values for some of the psychological sub-scales do limit our ability to interpret these findings. For example, the difference in the treatment control sub-scale between the attenders and non-attenders may be a chance finding given the low internal consistency of this measure. The STAI anxiety questionnaire measures state rather than trait anxiety i.e. the person's anxiety at the time rather than their general level of anxiety. An assessment of trait anxiety might have been useful in confirming that raised levels of anxiety were not usual for those in whom it was observed (and thus were associated with the screening process). However, a sufficiently large number of participants were distributed at random into the groups, and therefore any unknown confounders (including trait anxiety) can be assumed to be evenly distributed between the groups, along with the known confounders included in Table 1.

## Conclusion

This paper describes the first individually-randomised controlled trial to assess the psychological impact of screening for diabetes at six weeks. Evidence from this study suggests that one-off screening in general practice is feasible and acceptable. However, in contrast to previous studies, our data imply that being invited to screening may be associated with increased anxiety six weeks later, particularly among those given a new diagnosis. While increased anxiety may not be an entirely negative consequence, it merits further consideration in order to optimise the balance of benefits and harms of any future screening programme.

## Abbreviations

STAI: (Short-form Spielberger State Anxiety Inventory); IPQ: (Illness Perception Questionnaire); ADDITION: (**A**nglo-**D**anish-**D**utch study of **I**ntensive **T**reatment **i**n Pe**o**ple with Scree**n **Detected Diabetes in Primary Care)

## Competing interests

The authors declare that they have no competing interests.

## Authors' contributions

This paper was written on behalf of the *ADDITION-Cambridge *study group. PP performed the data collection and statistical analysis and drafted the manuscript. RKS contributed to the writing of the manuscript. ATP contributed to the analysis and helped to draft the manuscript. SJG conceived the study and supervised the design, coordination and data analysis and helped to draft the manuscript. All authors read and approved the final manuscript

## Pre-publication history

The pre-publication history for this paper can be accessed here:


